# Temporal trends in the clinical presentation of Graves’ orbitopathy: a single–center retrospective study

**DOI:** 10.1007/s40618-024-02332-3

**Published:** 2024-03-15

**Authors:** R. Le Moli, A. Naselli, F. Lo Giudice, G. Costanzo, F. Frasca, A. Belfiore

**Affiliations:** Department of Clinical and Experimental Medicine, Endocrinology Unit, Garibaldi Nesima Hospital, University of Catania, Via Palermo 636, 95125 Catania, Italy

**Keywords:** Graves, Ophthalmopathy, Clinical presentation, Severity, Hyperthyroidism

## Abstract

**Purpose:**

Graves’ ophthalmopathy (GO) is an autoimmune disease that affects orbital soft tissues and represents the most common extrathyroidal manifestation of Graves’ disease (GD). The European Group of Graves’ Ophthalmopathy (EUGOGO) has attempted to shed light on the European epidemiological picture of GO, suggesting that GO in newly diagnosed patients in recent years has a trend towards a less severe clinical presentation. There are no studies that focus this issue on the population of our area; we aimed to evaluate the trend of GO clinical presentation in our outpatient clinic through an observation period of 10 years.

**Methods:**

We compared 55 consecutive patients, 11 males (F) and 44 females (M), who came to our observation from January 2005 to December 2006 [Group 1 (G1)], with 56 patients, 15 males, and 41 females, who were referred to us from 2015 to 2016 [Group 2 (G2)]. We studied the following putative predictors of GO presentation and severity: thyroid function, smoking, diabetes, hypercholesterolemia, time from GO diagnosis to referral to our thyroid centre (TGOD), sex and age.

**Results:**

GO severity was significantly reduced in G2 vs. G1 (*p* = 0.04). TGOD ≥ 3 months was related to clinical characteristics of GO (severity and Clinical Activity Score ≥ 4) and was an independent predictor of GO severity (*p* = 0.01). The other variables evaluated had no independent effects.

**Conclusions:**

We found that GO severity at presentation was significantly reduced over a ten-year observation period (2005–2006 vs. 2015–2016) in GO patients referred to our tertiary thyroid centre. TGOD ≥ 3 months was an independent predictor of GO severity.

## Introduction

Graves’ ophthalmopathy (GO) is an autoimmune disease that affects orbital soft tissues and represents the most common extrathyroidal manifestation of Graves’ disease (GD). Thyroid stimulating hormone receptor (TSHR) is believed to be the autoantigen shared between thyroid and orbit tissues [1–6]. There are several clinical variants of GO, which accounts for the lack of a clear epidemiological picture. In its more severe forms, GO has a major impact on the social sphere of the patient, as some clinical manifestations could be disfiguring and disabling, interfering with the subject’s daily life, work, and activities and consequently reducing quality of life (QoL) [7–9]. Unfortunately, some literature studies identified patients with GO through a registry; moreover, the estimates of the prevalence of GO are derived from a small number of epidemiological studies using different methodologies to diagnose GO. In recent times, the European Group of Graves’ Ophthalmopathy (EUGOGO) has attempted to shed light on the European epidemiological picture of GO, suggesting that GO in newly diagnosed patients in recent years has a trend towards a less severe clinical presentation. Indeed, it seems that the clinical phenotype of Graves’ disease is presently milder than that in the past [10–12]. Although it seems that severe forms of GO appear in general less frequently, there are no studies that focus on the population of our area. Considering that our centre is the only reference centre for GO in Sicily, we aimed to evaluate the temporal trend of the clinical presentation of GO in the eastern part of Sicily over the last decade.

## Aim

We aimed to evaluate the trend of GO clinical presentation in our outpatient clinic through an observation period of 10 years and to identify its predictive factors.

## Methods

### Patient selection

We selected 221 consecutive patients observed in our centre from January 2005 to December 2006 and from January 2015 to December 2016. We excluded 21 patients who had undergone surgical orbital decompression and 40 patients previously treated with oral or parenteral corticosteroids and/or radiation therapy, and 49 patients because they had been diagnosed with GO for more than 12 months at the first visit. Finally, we studied 111 patients with GD and untreated GO who was diagnosed within 12 months of the presentation of eye symptoms. We compared 55 patients, 11 males (F) and 44 females (M), who came to our observation from January 2005 to December 2006 [Group 1 (G1)], with 56 patients, 15 males, and 41 females, who were referred to us from 2015 to 2016 [Group 2 (G2)]. Thyroid function, smoking, diabetes, hypercholesterolemia, time from GO diagnosis to referral to our thyroid centre (TGOD), sex and age were analyzed as putative predictors of GO severity.

### GO clinical evaluation

The same trained endocrinologist and ophthalmologist examined the patients during both periods. GO was graded according to EUGOGO criteria. Quality of life was evaluated by a validated specific GO quality of life questionnaire (GO-QOL) [8]. Mild was defined as having no or mild impact on daily life; moderate to severe (MS) was defined as having a moderate impact on daily life and showing one or more of the following clinical signs: 2 mm or more above the normal value lid opening, moderate to severe soft tissue involvement, Hertel measurements 3 mm or more above 21, and constant or inconstant diplopia according to the Gorman score. GO was defined as severe (S) when dysthyroid optic neuropathy (DON) or severe corneal breakdown appeared. DON was defined clinically when visual acuity was less than 6/10 (by Snellen table) in almost one eye, more than two errors in the vision of tables of Ishihara were detected and the abnormalities of fundus oculi related to DON were present by ophthalmological inspection. GO was defined as asymmetric when the Hertel measurement or eyelid aperture was ≥2 mm or ≥3 mm, respectively, more in one orbit than in the other. GO activity was evaluated according to the seven-point Clinical Activity Score (CAS) and was defined as active when CAS ≥3.

### Analytical methods and metabolic evaluation criteria

Serum hormones were measured by microparticle enzyme immunoassay (Abbot AxSYM-MEIA) with interassay coefficients of variation of less than 10% over the analytical ranges of 1.7–46.0 pmol/L for FT3, 5.15–77.0 pmol/L for FT4 and 0.03–10.0 mU/L for TSH. The within-run and between-run precisions for FT3, FT4 and TSH assays showed coefficients of variation <5%. Thyrotropin receptor antibodies (TRAbs) were measured by a 3° generation assay (SELco TRAbs Human, Dahlewitz/Berlino (Germany). Diabetes was defined by fasting glycaemic values ≥126 mg/dl, impaired fasting glucose (IFG) ≥100 mg/dl and impaired glucose tolerance (IGT) ≥115 mg/dl, the diagnoses of Diabetes, IFG and IGT were validated by the evidence of fasting glycaemic values beyond the established threshold in more than one determination. Hypercholesterolemia was defined by LDL cholesterol ≥140 mg/dl calculated by the formula of Martin/Hopkins, which is reliable for a wide range of triglyceride values. Glycaemia and cholesterol plasmatic levels were evaluated by standard analytical methods.

### Statistical analysis

We used the SPSS package (IBM SPSS statistics for Windows, version 20 Armonk, (NY:IBM Corp) for statistical analysis. Data were available on the diagnosis of GD, the onset of GO symptoms, and the initiation of GO immunosuppressive therapy. Continuous variables were analysed by Student’s *t* test, nonparametric continuous variables were analysed by the Mann‒Whitney U test, and the chi-square test was used when appropriate. Moderate to severe and severe GO were analysed both singularly and as one group (MS + S). We also evaluated whether TGOD above 50° percentiles (TGOD ≥ 3 months) could be correlated with the clinical characteristics of GO. More specifically, we built two different binomial logistic regression models (M1 and M2). M1 was built by considering the effect of age, sex, GO severity and setting the two groups of GO patients (G1: 2005–2006 vs. G2: 2015–2016) as dependent variables. By M2 we analyzed the predictive role of TGOD ≥ 3 months, Hertel measurements, CAS, diplopia, and belonging to G1 or G2, that we set as covariates respect to GO severity that we set as the dependent variable. The chosen significance level was *p* = 0.05.

## Results

The characteristics of the patients studied are depicted in Table 1.

**Table 1:** Characteristics of the 111 GO patients in the study

	2005–2006 (G1)	2015–2016 (G2)	*P*
Number of patients with GO (F/M)	55 (44/11)	56 (41/15)	ns
Thyroid function (number of Eu/Hypo/Hyper)	4/1/50	2/0/54	
GO F/M ratio	4/1	3/1	ns
Smokers *n*, (%)	21 (38.2%)	25 (44.6%)	ns
Female smokers *n*, (%)	15 (34.1%)	18 (43.9%)	ns
Male smokers *n*, (%)	6 (54.5%)	9 (60%)	ns
Female age at GO presentation (years)	42.9 ± 9.6	45.9 ± 12.1	ns
Male age at GO presentation (years)	45.1 ± 11.9	50.1 ± 12.2	ns
Diabetes-IFG-IGT	2–3-1	2–3-2	ns
LDLc > 140 mg/dl (number)	10	11	ns
TRAb (UI/L)	7.2 (2.8–22.5)	4.8 (1.0–21.1)	ns

*GO* Graves’ Ophthalmopathy, *LDLc* Low Density Lipoprotein Cholesterol, *IGT* Impaired Glucose Tolerance, *IFG* Impaired Fasting Glucose, *TRAb *Thyroid Receptor Antibody

### Sex and age of patients at GO presentation

GO clinical presentation showed no sex differences over time when we compared the two observation periods. The age at presentation was similar in male and female GO patients and showed no significant differences between the two evaluation periods: 45.9 ± 12.1 vs. 42.9 ± 9.6 years, G2 vs. G1 (females, *p* = ns) and 50.1 ± 12.1 vs. 45.1 ± 12.9 years, G2 vs. G1 (males, *p* = ns) (Table 1).

### Thyroid function

Thyroid function at presentation was not different between the two groups. All patients were treated with methimazole doses ranging from 10 to 30 mg daily. The majority of the study patients had clinical or subclinical hyperthyroidism, and this proportion was similar in both groups (Table 1). Based on geographical residence, dietary habits and medications, we assumed that iodine and selenium intake were similar between the two groups of patients.

### Smoking status, metabolic parameters, immunity markers

The percentage of GO smokers was similar between the two different groups of patients: 38.2 vs. 44.6 (G1 vs. G2, *p* = ns); however, the percentage of female smokers showed an incremental trend through the studied periods, although it was not statistically significant: 34.1 vs. 43.9 (G1 vs. G2, *p* = ns). The presence of diabetes, impaired glucose tolerance and impaired fasting glycaemia were similar between the two groups as the GO patients with LDL cholesterol >140 mg/dl. TRAbs levels and their rate of positivity were not significantly different between the two groups of patients (Table 1).

### GO severity and activity

**Fig. 1:**
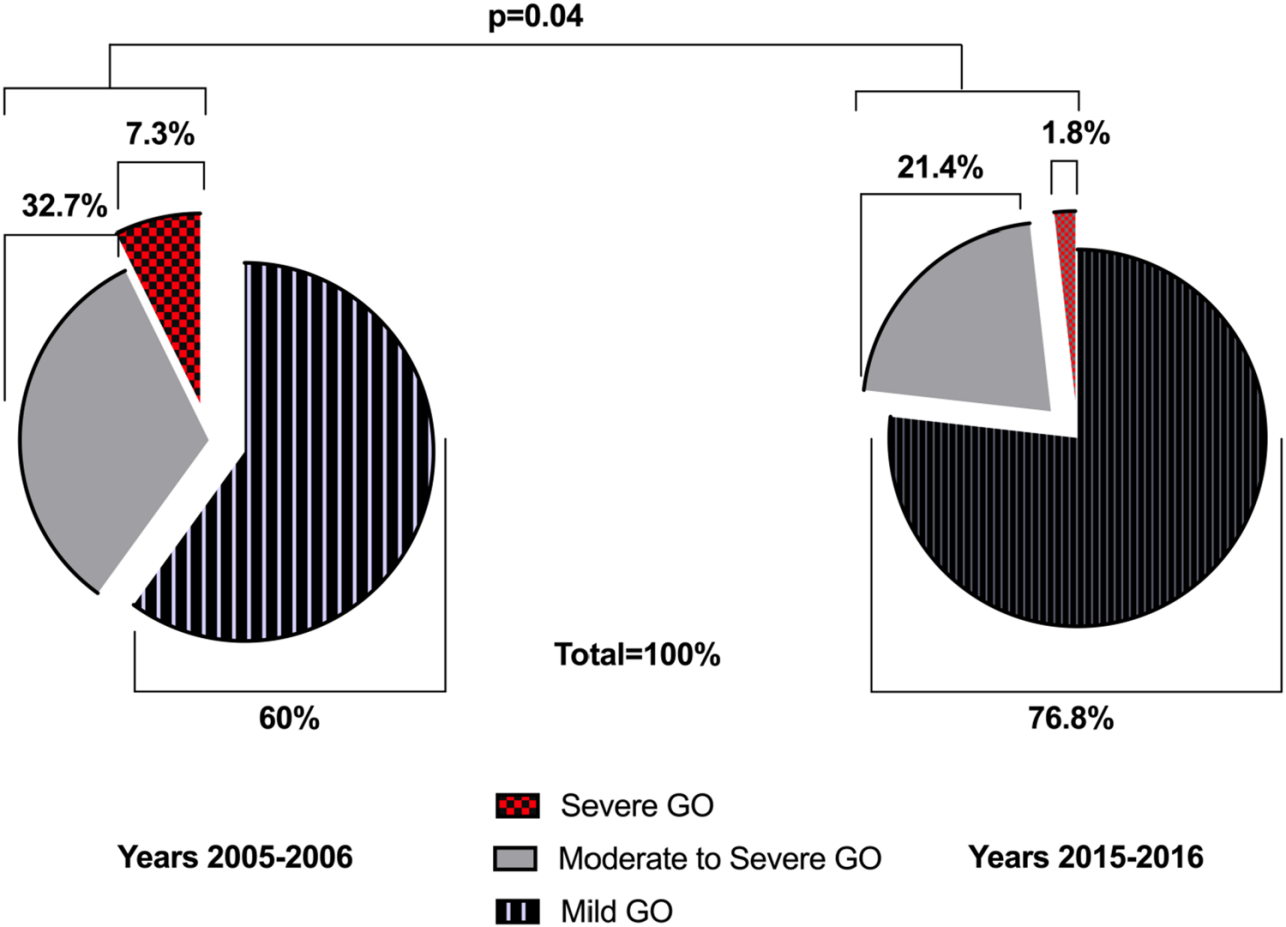
Clinical phenotypes in GO presentation during the two periods of observation (2005–2006 vs. 2015–2016)

One patient in G2 (1.8%) vs. 4 patients in G1 (7.3%) had sight-threatening GO because of corneal involvement and clinical optic neuropathy. Nine patients in G2 (16.1%) vs. 12 patients in G1 (21.8%) had moderate to severe GO. Two patients in G1 and 1 patient in G2 had very light involvement of the corneal surface but were considered to have moderate to severe GO. Forty-three patients in G2 (76.8%) vs. 33 patients in G1 (60.0%) had mild GO. Hertel measurements were not different between the two groups of patients: 19.5 ± 3.1 vs. 19.1 ± 3.2; (G2 vs. G1, *p* = ns). GO was asymmetric in 3 (5.4%) patients with G2 vs. 4 (7.3%) patients with G1 (*p* = ns). Diplopia evaluated according to Gorman score was present in 20 (35.7) vs. 23 (41.8) patients (G2 vs. G1, *p* = ns). GO was active in 26 (46.4%) vs. 17 (30.9%) patients, (CAS > 3; G2 vs. G1, *p* = ns). GO was very active in 2 (3.6%) vs. 5 (9.1%) patients (CAS > 4, G2 vs. G1, *p* = 0.09). A less severe GO clinical presentation in patients of G2 vs. G1 was evidenced, but it was not statistically significant. In contrast, the trend became statistically significant when moderate to severe and sight-threatening GO were considered together, 13 (23.2%) vs. 22 (40.0%) patients, respectively, G2 vs. G1, *p* = 0.04. TGOD and therapies offered were not different between the two groups of patients (Table 2; Fig. 1).

**Table 2:** Clinical characteristics of GO in the two periods studied

	2005–2006 (G1) 55	2015–2016 (G2) 56	*P*
S GO *n*, (%)	4 (7.3)	1 (1.8)	0.2
MS GO *n*, (%)	18 (32.7)	12 (21.4)	0.3
MS + S GO *n*, (%)	22 (40.0)	13 (23.2)	0.04
Mild GO *n*, (%)	33 (60.0)	43 (76.8)	0.5
CAS	2.0 (0–4)	2.1 (0–4)	0.7
Corneal involvement *n*, (%)	6 (10.9)	2 (3.6)	0.2
Dysthyroid optic neuropathy (DON) *n*, (%)	4 (7.3)	1 (1.8)	0.2
Active GO CAS ≥ 3 *n*, (%)	17 (31)	26 (46)	0.5
Active GO CAS ≥ 4 *n*, (%)	5 (9.1)	2 (3.6)	0.09
Asimmetrical GO *n*, (%)	4 (7.2)	3 (5.3)	0.2
Hertel (mm)	19.1 ± 3.2	19.5 ± 3.1	0.4
Diplopia *n*, (%)	23 (41.8)	20 (35.7)	0.4
TGOD (months)	3.6 ± 1.8	3.2 ± 1.7	0.2
Therapy (oS/ivS/Radio/Radio + ivS)	3/10/1/3	1/6/1/4	

*Go S (n-%)* Sight-threatening GO(n-%), *GO MS (n-%)* Moderate to severe form of GO, *GO MS + GO S (n-%)* sum of moderate-severe and severe forms of GO, *CAS* Clinic Activity Score, *Hertel(mm)* exophthalmos measurement, *TGOD* time from GO diagnosis to referral to our centre (months), *Therapy (oS/ivS/Radio/Radio + ivS)* therapy with oral steroids or intravenous steroids or Radio therapy or Radio therapy + intravenous steroids

### Correlation of TGOD with clinical characteristics of GO; Binomial logistic regression analysis, M1 and M2 models

The mean number of months elapsed from time from GO diagnosis to referral to our centre (TGOD) was similar in the two groups (3.6 ± 1.8 vs. 3.2 ± 1.7, in G1 and G2, respectively) (Table 2) although the number of GO patients with referral time ≥3 months (TGOD ≥ 3 months [≥50° percentiles]) was correlated with the severity of GO and with the CAS ≥ 4 (Chi-square: *p* = 0.04 and *p* = 0.02 respectively); CAS was significantly increased in patients with TGOD ≥ 3 months (Man Withney-U: *p* = 0.03).

According to M1 binomial logistic regression model the risk for GO severity was significantly related to the two different periods studied (G1 [2005–2006] and G2 [2015–2016]) and was reduced in G2 vs. G1 (*p* = 0.03) confirming the data of univariate analysis. Age and sex had no effect in the two different periods studied (Table 3). By model M2, we found that belonging to G1 was an independent predictor of increased risk to have more severe GO (*p* = 0.01). Moreover, TGOD ≥ 3 months independently increased the risk for more severe GO (*p* = 0.01). Hertel measurement as expected was another predictor of GO severity (*p* < 0.001) (Table 4).

**Table 3:** Binomial logistic regression analysis (model M1) comparing GO clinical characteristics in the two patient groups

	G1 (2005–2006) vs. G2 (2015–2016)			
	Wald	Exp (B)	95% CI	*P*
Age	0.34	0.99	0.95–1.02	0.5
Sex	1.29	1.73	0.67–4.49	0.2
GO severity	4.71	2.61	1.12–6.24	0.03

*GO severity* Graves’ ophthalmopathy severity including moderate to severe and severe GO

**Table 4:** Binomial logistic regression analysis (model M2) of GO severity in relation to the two groups of patients, TGOD and clinical variables

	GO severity			
	Wald	Exp (B)	95% CI	*P*
G1 (2005–2006)–G2 (2015–2016)	6.09	4.38	1.35–14.16	0.01
TGOD ≥ 3* months*	6.47	5.54	1.48–20.75	0.01
CAS	3.42	1.71	0.97–3.03	0.06
Hertel	15.01	1.64	1.27–2.11	0.001
Diplopia	2.61	0.41	0.12–1.21	0.2

*TGOD ≥ 3 mo.* time from GO diagnosis to our center referral ≥ 3 months, *CAS* seven points clinical activity score, *G1* group 1, *G2* group 2

## Discussion

GO is a rather complex disorder because the clinical presentation is very different from patient to patient. The GO clinical phenotype is mild at presentation for approximately 80% of cases, moderate to severe for 15% and sight threatening for less than 2%. Approximately 2% of mild cases turn into moderate to severe GO and approximately 1% into severe GO, most of these changes occur 6–12 months after the diagnosis of GO [9]. It has been hypothesized that there is a declining trend in the clinical severity of GO at presentation in European populations. Very recently, the European Group of Graves’ ophthalmopathy (EUGOGO) published the PREGO III study [11, 12], and the authors of this study compared the data of 269 GO patients evaluated in 2012 from 15 different European centres with 432 GO patients evaluated in 2019 from 41 centres. The authors stated that GO severity was significantly decreased in 2019 with respect to 2012 and concluded that “These changes might reflect a general increased awareness of EUGOGO centres and activity among general physicians/endocrinologists and patient organizations”. We compared the clinical presentation of 55 (G1) GO patients who were referred to our centre from 2005 to 2006 with that of 56 (G2) GO patients who were observed from 2015 to 2016. We included only patients with GO presentation within 12 months of diagnosis of GD, and studied the following putative predictors of GO presentation and severity: thyroid function, smoking, diabetes, hypercholesterolemia, TGOD, sex and age. Thyroid function is a very important variable that modulates GO activity and severity, and to normalize thyroid function is the first step to prevent GO presentation and/or exacerbation [13–15]. Hyperthyroidism causes oxidative stress that increases innate and adaptive immune responses in patients with GD, increasing the risk of GO presentation [16], although hypothyroidism also increases the risk of GO presentation and exacerbation by TSHR overactivation [17]. However, thyroid function was similar between the two different groups of patients with GO studied. Smoking is the most recognized risk factor for GO presentation and exacerbation since smokers have more severe GO than nonsmokers [18]. Smoking, both directly and indirectly, plays a role in GO clinical presentation and evolution. Chronic hypoxia could redirect local macrophage function and orbit fibroblast proliferation by increasing Ipossic-Factor-1 (IF-1) and promoting some different grades of inflammation. Smoke increases the level of hyaluronic acid and glycosaminoglycan production by orbit fibroblasts, and this action is synergistic with interleukin-1 (IL-1) activity. Moreover, smoking could affect GO by direct trauma from heat through the medial orbit wall, which is very thin. According to clinical evidence, smoking is considered the most crucial factor for GO presentation and exacerbation [19–22]; some authors state that there seems to be a trend towards a decline in progression of GO, possibly due to better control of cigarette smoking in the past years. However, decline of smoking is not linear among the different European populations and it is related to education of peoples, smoking in fact has been growing between people with basic education and among female of developing countries [23]. In our study, the habit of smoking was not different between males and females or between the studied periods. We noted an incremental trend for smoking in females during the last period (2015–2016 vs. 2005–2006); however, it was not statistically significant although the lack of effect noted may be due to the small sample of patients studied, as the trend in smoking habits in Italy is not declining.

Recently, type 2 diabetes (DMT2) and/or cholesterol have been proven to be risk factors for GO presentation and severity. DMT2 and/or hypercholesterolemia cause a systemic inflammatory state that adds to the GO immunological process and contributes to the remodelling of orbital tissues. Elevated LDLc levels increase the influx of free fatty acids into the liver, causing the production of reactive oxygen radical species (ROS) and interleukin-6 (IL-6). IL-6 activity within the orbital environment can promote the secretion of insulin growth Factor 1 (IGF-1), improving the expansion of soft orbital tissues [24–27]. We did not find differences in the LDLc or glucose levels of the GO patients studied. We did not observe any difference in sex or age between the two groups of patients. The role of iodine supplementation and GO severity has been evaluated in the Scandinavian population by Laurberg et al. [28]. These authors concluded that GO clinical presentation is not related to the iodine intake of the population. We studied a population from the eastern part of Sicily. Iodine supplementation in that area started in 1996, and according to the last studies by *ISTISAN* [29], urinary iodine excretion of the Sicilian population was normalized during the last 15 years.

Selenium (SE) intake is another factor related to the severity of GO. SE is an essential nutrient for the synthesis of selenocysteine, which is part of selenoproteins, mostly enzymes, in which SE acts as an antioxidant. The increase in the generation of oxygen free radicals plays a pathogenic role in GO. SE reduces oxidative stress and cytokine levels, decreasing orbital fibroblast proliferation in the orbit of GO patients. Some studies have shown that SE levels are lower in GO patients; moreover, SE supplementation is effective in preventing GO presentation and exacerbation [30, 31]. SE supplementation was not in use until 2005, and in any case, no patients were given SE before our clinical evaluation. We conclude that all putative predictors of GO presentation and severity were similar between the two groups of GO patients, as evidenced by univariate analysis. The TGOD was not different in G1 respect G2 at univariate analysis, however, when we studied all the patients with TGOD ≥ 3 months (TGOD duration in months ≥50° percentiles) we found a correlation with the severity of GO and with CAS ≥ 4. Moreover, the number of patients with CAS ≥ 4 was significantly increased in patients with TGOD ≥ 3 months. We finally evaluated sex, age, GO severity, respect the two different period of GO evaluation (G1 [2005–2006] vs. G2 [2015–2016]) by a binomial logistic regression analysis model (M1). This model indicated that GO severity at presentation was significantly different in the two different periods chosen (*p* = 0.03); age and sex had no significant effect (Table 3). To better understand the role of TGOD and of the two different period studied on GO severity we build a second regression model (M2) with some putative predictors of GO severity set as covariates: number of patients with TGOD ≥ 3 months (TGOD ≥ 50° percentile), G1 vs. G2, Hertel measurements, CAS and diplopia set as covariates and GO severity set as the dependent variable; this model evidenced the independent role of the two different period studied (G1 [2005–2006] vs. G2 [2015–2016]) and TGOD ≥ 3 months as predictors of GO severity (both *p* = 0.01). As expected Hertel measurement was also an independent predictor of GO severity (*p* = 0.03) (Table 4).

The present study indicates that GO more frequently occurs as a less severe form in the last decade and suggests that TGOD ≥ 3 months is an independent predictor of the clinical characteristics of GO at presentation. These data also suggest that a closer interaction between general medicine doctors, endocrinologists and ophthalmologists should be mandatory in the management of thyroid and orbital disease [12]. This interpretation, however, should be supported by prospective studies, which are very difficult to perform, as GO is almost a rare disease. The main strength of our study is the standardized methodology used to select GO patients. All patients were seen at our outpatient clinics for GO, which is the only EUGOGO centre recognized in Sicily and the same trained endocrinologist and ophthalmologist visited all patients in the study. The major limitation of our study is represented by the limited number of patients.

## Conclusions

We found that GO severity at presentation was significantly reduced over a ten-year observation period (2005–2006 vs. 2015–2016) in GO patients referred to our tertiary thyroid centre (EUGOGO recognized centre) before starting any specific treatment. The two different periods of observation and the TGOD ≥ 3 months were independent predictors of GO severity. However, these results should be further evaluated by prospective studies aimed at identifying the relevant variables involved in changes in the clinical presentation of GO over time.

## Data Availability

The data associated with this paper are available at the Department of Clinical and Experimental Medicine of University of Catania.

## References

[1] Bahn RS (2010) Grave’s ophthalmopathy. N Engl J Med 362:726–738. 10.1056/NEJMra0905750 20181974 10.1056/NEJMra0905750 PMC3902010

[2] Lazarus JH, Marino M (2010) Orbit-thyroid relationship. In: Wiersinga WM, Kahaly GJ (eds) Graves’ orbitopathy—a multidisciplinary approach—question and answers. Karger, Basel, pp 26–32. 10.1159/000320425

[3] Rapoport B, McLachlan SM (2007) The thyrotropin receptor in Graves’ disease. Thyroid 17:911–922. 10.1089/thy.2007.0170 17822379 10.1089/thy.2007.0170

[4] Krieger CC, Place RF, Bevilacqua C, Marcus-Samuels B, Abel BS, Skarulis MC, Kahaly GJ, Neumann S, Gershengorn MC (2016) TSH/IGF-1 receptor cross talk in Graves’ ophthalmopathy pathogenesis. J Clin Endocrinol Metab 101:2340–2347. 10.1210/jc.2016-1315 27043163 10.1210/jc.2016-1315 PMC4891793

[5] Smith TJ, Janssen JAMJL (2017) Building the case for insulin-like growth factor receptor-I involvement in thyroid-associated ophthalmopathy. Front Endocrinol 7:1–8. 10.3389/fendo.2016.00167 10.3389/fendo.2016.00167 PMC5206614 28096798

[6] Krieger CC, Neumann S, Gershengorn MC (2020) TSH/IGF1 receptor crosstalk: mechanism and clinical implications. Pharmacol Ther 209:107502. 10.1016/j.pharmthera.2020.107502 32061922 10.1016/j.pharmthera.2020.107502 PMC7187798

[7] Perros P, Hegedüs L, Bartalena L, Marcocci C, Kahaly GJ, Baldeschi L, Salvi M, Lazarus JH, Eckstein A, Pitz S, Boboridis K, Anagnostis P, Ayvaz G, Boschi A, Brix TH, Currò N, Konuk O, Marinò M, Mitchell AL, Stankovic B, Törüner FB, Von Arx G, Zarković M, Wiersinga WM (2017) Graves’ orbitopathy as a rare disease in Europe: a European Group on Graves’ Orbitopathy (EUGOGO) position statement. Orphanet J Rare Dis 12:4–9. 10.1186/s13023-017-0625-1 28427469 10.1186/s13023-017-0625-1 PMC5397790

[8] Bartalena L, Kahaly GJ, Baldeschi L, Dayan CM, Eckstein A, Marcocci C, Marinò M, Vaidya B, Wiersinga WM (2021) The 2021 European Group on Graves’ orbitopathy (EUGOGO) clinical practice guidelines for the medical management of Graves’ orbitopathy. Eur J Endocrinol 185:G43–G67. 10.1530/EJE-21-0479 34297684 10.1530/EJE-21-0479

[9] Tanda ML, Piantanida E, Liparulo L, Veronesi G, Lai A, Sassi L, Pariani N, Gallo D, Azzolini C, Ferrario M, Bartalena L (2013) Prevalence and natural history of Graves’ orbitopathy in a large series of patients with newly diagnosed Graves’ hyperthyroidism seen at a single center. J Clin Endocrinol Metab 98:1443–1449. 10.1210/jc.2012-3873 23408569 10.1210/jc.2012-3873

[10] Bartalena L, Masiello E, Magri F, Veronesi G, Bianconi E, Zerbini F, Gaiti M, Spreafico E, Gallo D, Premoli P, Piantanida E, Tanda ML, Ferrario M, Vitti P, Chiovato L (2016) The phenotype of newly diagnosed Graves’ disease in Italy in recent years is milder than in the past: results of a large observational longitudinal study. J Endocrinol Invest 39:1445–1451. 10.1007/s40618-016-0516-7 27465670 10.1007/s40618-016-0516-7

[11] Perros P, Zarkovi M, Azzolini C, Ayvaz G, Baldeschi L, Bartalena L, Boschi A, Bournaud C, Brix TH, Covelli D, Iri S, Daumerie C, Eckstein A, Fichter N, Führer D, Hegedüs L, Kahaly GJ, Konuk O, Lareida J, Lazarus J, Leo M, Mathiopoulou L, Menconi F, Morris D, Okosieme O, Orgiazzi J, Pitz S, Salvi M, Vardanian-Vartin C, Wiersinga W, Bernard M, Clarke L, Currò N, Dayan C, Dickinson J, Knezevi M, Lane C, Marcocci C, Marinò M, Möller L, Nardi M, Neoh C, Pearce S, Von Arx G, Törüner FB (2015) PREGO (presentation of Graves’ orbitopathy) study: changes in referral patterns to European Group on Graves’ Orbitopathy (EUGOGO) centres over the period from 2000 to 2012. Br J Ophthalmol 99:1531–1535. 10.1136/bjophthalmol-2015-306733 25953846 10.1136/bjophthalmol-2015-306733

[12] Schuh A, Ayvaz G, Baldeschi L, Baretić M, Bechtold D, Boschi A, Brix TH, Burlacu M-C, Ciric J, Covelli D, Currò N, Donati S, Eckstein AK, Fichter N, Führer D, Horn M, Jabłońska-Pawlak A, Juri Mandić J, Kahaly GJ, Konuk O, Langbein A, Lanzolla G, Marcocci C, Marinò M, Miśkiewicz P, Beleslin BN, Pérez-Lázaro A, Pérez-López M, Ponto KA, Quinn A, Rudofsky G, Salvi M, Schittkowski MP, Tanda ML, Toruner F, Vaidya B, Hintschich CR (2023) Presentation of Graves’ orbitopathy within European Group On Graves’ Orbitopathy (EUGOGO) centres from 2012 to 2019 (PREGO III). Br J Ophthalmol 108:294–300. 10.1136/bjo-2022-322442 10.1136/bjo-2022-322442 PMC10850632 36627174

[13] Bartalena L (2011) The dilemma of how to manage Graves’ hyperthyroidism in patients with associated orbitopathy. J Clin Endocrinol Metab 96:592–599. 10.1210/jc.2010-2329 21190983 10.1210/jc.2010-2329

[14] Wiersinga W, Žarković M, Bartalena L, Donati S, Perros P, Okosieme O, Morris D, Fichter N, Lareida J, Von Arx G, Daumerie C, Christina Burlacu M, Kahaly G, Pitz S, Beleslin B, Ćirić J, Ayvaz G, Konuk O, Töröner FB, Salvi M, Covelli D, Curro N, Hegedös L, Brix T (2018) Predictive score for the development or progression of Graves’ orbitopathy in patients with newly diagnosed Graves’ hyperthyroidism. Eur J Endocrinol 178:635–643. 10.1530/EJE-18-0039 29650691 10.1530/EJE-18-0039

[15] Träisk F, Tallstedt L, Abraham-Nordling M, Andersson T, Berg G, Calissendorff J, Hallengren B, Hedner P, Lantz M, Nyström E, Ponjavic V, Taube A, Törring O, Wallin G, Åsman P, Lundell G, Lindstedt G, Michanek A, Norrsell K, Valdemarsson S, Garkavij M, Tennvall J, Widmark H, Stigmar G, Arwidi Å, Bjelkengren G, Hemdahl B, Jönsson H, Becker C, Freyschuss B, Hoffstedt J, Tullgren O, Wahrenberg H, Wennlund A, Röjdmark S, Sääf M, Thorén M, Hamberger B, Blomgren H, Hilding C, Hjelm Skog AL (2009) Thyroid-associated ophthalmopathy after treatment for Graves’ hyperthyroidism with antithyroid drugs or iodine-131. J Clin Endocrinol Metab 94:3700–3707. 10.1210/jc.2009-0747 19723755 10.1210/jc.2009-0747

[16] Žarković M (2012) The role of oxidative stress on the pathogenesis of Graves’ disease. J Thyroid Res 2012:302537. 10.1155/2012/302537 22175033 10.1155/2012/302537 PMC3235898

[17] Bartalena L, Baldeschi L, Boboridis K, Eckstein A, Kahaly GJ, Marcocci C, Perros P, Salvi M, Wiersinga WM (2016) The 2016 European Thyroid Association/European Group on Graves’ Orbitopathy guidelines for the management of Graves’ orbitopathy. Eur Thyroid J 5:9–26. 10.1159/000443828 27099835 10.1159/000443828 PMC4836120

[18] Manji N, Carr-Smith JD, Boelaert K, Allahabadia A, Armitage M, Chatterjee VK, Lazarus JH, Pearce SHS, Vaidya B, Gough SC, Franklyn JA (2006) Influences of age, gender, smoking, and family history on autoimmune thyroid disease phenotype. J Clin Endocrinol Metab 91:4873–4880. 10.1210/jc.2006-1402 16968788 10.1210/jc.2006-1402

[19] Wiersinga WM (2013) Smoking and thyroid. Clin Endocrinol (Oxf) 79:145–151. 10.1111/cen.12222 23581474 10.1111/cen.12222

[20] Bartalena L, Marcocci C, Tanda ML, Manetti L, Dell’Unto E, Bartolomei MP, Nardi M, Martino E, Pinchera A (1998) Cigarette smoking and treatment outcomes in Graves ophthalmopathy. Ann Intern Med 129:632–635. 10.7326/0003-4819-129-8-199810150-00010 9786811 10.7326/0003-4819-129-8-199810150-00010

[21] Cawood TJ, Moriarty P, O’Farrelly C, O’Shea D (2007) Smoking and thyroid-associated ophthalmopathy: a novel explanation of the biological link. J Clin Endocrinol Metab 92:59–64. 10.1210/jc.2006-1824 17047020 10.1210/jc.2006-1824

[22] Kau HC, Wu SB, Tsai CC, Liu CJL, Wei YH (2016) Cigarette smoke extract-induced oxidative stress and fibrosis-related genes expression in orbital fibroblasts from patients with Graves’ ophthalmopathy. Oxid Med Cell Longev 2016:4676289. 10.1155/2016/4676289 27340508 10.1155/2016/4676289 PMC4909929

[23] Tehrani H, Mahdizadeh M, Peyman N, Gholian-Aval M, Charoghchian Khorasani E, Jafari A (2022) Exploration factors on smoking among female adolescents based on the viewpoints of Iranian adolescent girls. BMC Womens Health 22:1–8. 10.1186/s12905-022-01791-1 35650621 10.1186/s12905-022-01791-1 PMC9158312

[24] Le Moli R, Muscia V, Tumminia A, Frittitta L, Buscema M, Palermo F, Sciacca L, Squatrito S, Vigneri R (2015) Type 2 diabetic patients with Graves’ disease have more frequent and severe Graves’ orbitopathy. Nutr Metab Cardiovasc Dis 25:452–457. 10.1016/j.numecd.2015.01.003 25746910 10.1016/j.numecd.2015.01.003

[25] Lanzolla G, Vannucchi G, Ionni I, Campi I, Sileo F, Lazzaroni E, Marinò M (2020) Cholesterol serum levels and use of statins in Graves’ orbitopathy: a new starting point for the therapy. Front Endocrinol 10:1–8. 10.3389/fendo.2019.00933 10.3389/fendo.2019.00933 PMC6987298 32038490

[26] Naselli A, Moretti D, Regalbuto C, Arpi ML, Lo Giudice F, Frasca F, Belfiore A, Le Moli R (2020) Evidence that baseline levels of low-density lipoproteins cholesterol affect the clinical response of Graves’ ophthalmopathy to parenteral corticosteroids. Front Endocrinol 11:1–10. 10.3389/fendo.2020.609895 10.3389/fendo.2020.609895 PMC7784376 33414766

[27] Neag EJ, Smith TJ (2022) 2021 update on thyroid-associated ophthalmopathy. J Endocrinol Invest 45:235–259. 10.1007/s40618-021-01663-9 34417736 10.1007/s40618-021-01663-9 PMC9455782

[28] Laurberg P, Berman DC, Pedersen IB, Andersen S, Carlé A (2012) Incidence and clinical presentation of moderate to severe Graves’ orbitopathy in a Danish population before and after iodine fortification of salt. J Clin Endocrinol Metab 97:2325–2332. 10.1210/jc.2012-1275 22518849 10.1210/jc.2012-1275 PMC3387399

[29] Olivieri A, Vitti P (2014) Rapporti ISTISAN 14/6. ISSN, 1123–3127

[30] Marinò M, Menconi F, Dottore GR, Leo M, Marcocci C (2018) Selenium in Graves hyperthyroidism and orbitopathy. Ophthal Plast Reconstr Surg 34:S105–S110. 10.1097/IOP.0000000000001136 29933354 10.1097/IOP.0000000000001136

[31] Marcocci C, Kahaly GJ, Krassas GE, Bartalena L, Prummel M, Stahl M, Altea MA, Nardi M, Pitz S, Boboridis K, Sivelli P, von Arx G, Mourits MP, Baldeschi L, Bencivelli W, Wiersinga W (2011) Selenium and the course of mild Graves’ orbitopathy. N Engl J Med 364:1920–1931. 10.1056/nejmoa1012985 21591944 10.1056/nejmoa1012985

